# Electro-Mechanical Characterization and Modeling of a Broadband Piezoelectric Microgenerator Based on Lithium Niobate

**DOI:** 10.3390/s24092815

**Published:** 2024-04-28

**Authors:** Namanu Panayanthatta, Giacomo Clementi, Merieme Ouhabaz, Samuel Margueron, Ausrine Bartasyte, Mickael Lallart, Skandar Basrour, Roberto La Rosa, Edwige Bano, Laurent Montes

**Affiliations:** 1 Centre for Radiofrequencies, Optics and Micro-Nanoelectronics in the Alps, University Grenoble Alpes, University Savoie Mont Blanc, CNRS, Grenoble INP, CROMA, 38000 Grenoble, France; 2 CNRS (UMR 6174), ENSMM, FEMTO-ST Institute, University of Bourgogne Franche-Comté, 26 Rue de l’Epitaphe, 25030 Besançon, Franceausrine.bartasyte@femto-st.fr (A.B.); 3 Institut Universitaire de France (IUF), 75013 Paris, France; 4 CNRS, Grenoble INP, TIMA—University Grenoble Alpes, 38000 Grenoble, France; 5 INSA-Lyon, LGEF EA682, University Lyon, 69621 Lyon, France; skandar.basrour@univ-grenoble-alpes.fr; 6 STMicroelectronics, Stradale Primosole 50, 95121 Catania, Italy; roberto.larosa@st.com

**Keywords:** piezoelectric energy harvester, lithium niobate, lead-free piezoelectrics, IoT, broadband energy harvesting

## Abstract

Vibration energy harvesting based on piezoelectric transducers is an attractive choice to replace single-use batteries in powering Wireless Sensor Nodes (WSNs). As of today, their widespread application is hindered due to low operational bandwidth and the conventional use of lead-based materials. The Restriction of Hazardous Substances legislation (RoHS) implemented in the European Union restricts the use of lead-based piezoelectric materials in future electronic devices. This paper investigates lithium niobate ($LiNbO_{3}$) as a lead-free material for a high-performance broadband Piezoelectric Energy Harvester (PEH). A single-clamped, cantilever beam-based piezoelectric microgenerator with a mechanical footprint of 1 cm^2^, working at a low resonant frequency of 200 Hz, with a high piezoelectric coupling coefficient and broad bandwidth, was designed and microfabricated, and its performance was evaluated. The PEH device, with an acceleration of 1 g delivers a maximum output RMS power of nearly 35 μW/cm^2^ and a peak voltage of 6 V for an optimal load resistance at resonance. Thanks to a high squared piezoelectric electro-mechanical coupling coefficient ($k^{2}$), the device offers a broadband operating frequency range above 10% of the central frequency. The Mason electro-mechanical equivalent circuit was derived, and a SPICE model of the device was compared with experimental results. Finally, the output voltage of the harvester was rectified to provide a DC output stored on a capacitor, and it was regulated and used to power an IoT node at an acceleration of as low as 0.5 g.

## 1. Introduction

In recent years, harvesting power from ambient vibrations has been widely studied to supply low-power wireless sensors or actuators used in “Internet of things” (IoT) networks [1,2] to replace single-use batteries that show significant limitations in terms of lifespan due to their self-discharge. Unfortunately, Moore’s law analogy does not apply to batteries, as the annual amelioration in battery capacity is only 8%. Further, frequent maintenance and battery replacement are undesirable in next-generation IoT systems due to cost and environmental issues. Hence, battery-free and energy-autonomous IoT devices, which generally require very low power, ranging from μW to mW to operate, are a key area of focus within the green IoT concept. In this context, piezoelectric Energy Harvesters (PEHs), which produce a voltage across electrodes in the application of strain, have gained attention for ambient vibration energy harvesting. PEHs, due to their high power density, conversion efficiency at the micro-scale, and simple design, can replace batteries or improve their operation lifetime. Currently, most high-performance PEHs are based on lead-based materials, essentially PZT [3,4,5], and there has been little effort to research and develop next-generation “green PEHs” with a high global (i.e., in terms of structure aspects) electro-mechanical coupling coefficient, $k^{2}$, despite the growing consensus in the electronic industry regarding the use of environmentally friendly materials such as lithium niobate [6] or lithium tantalate [7]. A relevant challenge related to PEHs concerns the narrow bandwidth of operating frequency, as the output voltage and power reduce drastically while shifting from the resonant frequency [8]. Coupled with the applied frequency of the mechanical force, the PEH intrinsically produces an AC output voltage. The voltage, thus, needs to be rectified in order to be effectively used to supply a wireless sensor node. Since the frequency of typical ambient environmental vibration sources is low (less than 300 Hz) and susceptible to variation to some extent, PEHs with low resonant frequencies and broadband operation capabilities are necessary for optimal realistic performance [9]. Several publications on piezoelectric microgenerators with various complex configurations, such as a membrane structure or multiple stacked piezoelectric layers [8,9,10,11], are proposed to induce non-linearity in a system and push towards a broader operating frequency regime. In [12,13], a low-threshold, nonlinear, bi-stable device that adopts a nonlinear, snap-through buckling configuration and two piezoelectric transducers that can scavenge energy in the frequency range from 0.5 Hz to 10 Hz has been proposed. However, such multi-degree-of-freedom approaches generally have limitations, such as reduced power density due to a low quality factor, *Q*. Recently, new interest has emerged in tuning the operating frequency of PEHs by exploiting the high electro-mechanical coupling effect [14]. Strongly coupled PEHs are, therefore, considered a solution to enlarge the frequency bandwidth. For PEHs with a large $k^{2}$, resonance splitting gives two well-separated output power peaks that extend the harvesting bandwidth (BW) [14,15]. Badel and Lefeuvre [16] showed that it is possible to widely tune the resonant frequency of highly coupled piezoelectric energy harvesters to achieve broadband energy harvesting. Such promising perspectives for broadband vibration energy harvesting are achieved through the design and fabrication of PEHs with a strong $k^{2}$. However, most highly coupled PEHs use lead-based materials such as PZT and PMN-PT. Nabavi and Zhang [17] proposed an AlN-based broadband MEMS piezoelectric energy harvester that utilizes multimodal and nonlinear mechanisms. Although AlN has received considerable attention as a replacement for lead-based materials, lead-free ”green PEHs” with a simple configuration, working at low ambient frequencies with a high structural coupling $k^{2}$, exhibiting broadband operations, are rarely reported, and recent works have demonstrated high-potential, single, thick lithium niobate layers in these applications [18,19].

This paper presents a highly coupled piezoelectric micro-generator with lead-free lithium niobate as an active piezoelectric material that operates at a low resonant frequency (200 Hz) with broadband operation of around 10% of the central frequency range. The characterization of the device’s performance, such as the output voltage and the produced power at an optimal load at various accelerations levels, is presented along with the extraction of internal parameters such as the global squared electro-mechanical coupling factor, $k^{2}$, and mechanical quality factor, $Q_{m}$. Further, the overall Mason’s electro-mechanical model of the device was derived from impedance analysis, and the results are compared with LT-SPICE simulations. An appropriate rectification circuit output was chosen to convert the AC voltage to DC based on extracted device parameters ($k^{2}Q_{m}$). The PEH was used to power a battery-free IoT sensor node with Bluetooth low-energy communication from vibrations with a minimum acceleration of 0.5 g.

## 2. PEH Design and Fabrication

**Figure 1:**
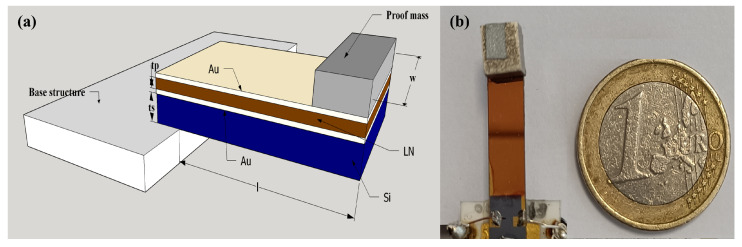
(**a**) Cross-sectional schematic. (**b**) Photograph of PEH device with a proof mass.

The global structural electro-mechanical coupling coefficient, $k_{t}$, is a key factor determining the performance of a PEH device with a cantilever structure. The coupling $k_{t}$ depends on the piezoelectric material coupling coefficient, $k_{m}$, Young’s modulus, and structural geometrical parameters, such as the length, *l*, the width, *w*, the thickness, $t_{p}$, and the $t_{s}$ of the piezoelectric layer and the substrate, respectively. $k_{t}$ can be improved as follows: (i) by using strongly coupled piezoelectric materials such as relaxor-based ferroelectric PMN-PT or crystals, PZT, KNN ceramics, lithium niobate crystals, and the like, and (ii) by optimizing the device geometry to homogenize the stress distribution in the piezoelectric layer, e.g., by adding a proof mass of high density [14]. The key step in highly coupled PEH fabrication based on $LiNbO_{3}$ is the bonding and thinning down of single-crystal $LiNbO_{3}$ films. To ensure a high electro-mechanical coupling and stress transmission, the thickness of the intermediate adhesive layer between Si and $LiNbO_{3}$ must be less than several microns. In addition, the thickness and initial warping/baw of the $LiNbO_{3}$ and Si wafer must be well controlled to thin the bulk piezoelectric material uniformly. Finally, the intermediate bonding layer should be conductive to act as a bottom electrode. The state-of-the-art technique reported in the literature for wafer bonding is the use of epoxy organic adhesive material as an intermediate bonding layer to bond the crystal to the silicon wafer [14]. However, the glue thickness is difficult to accurately control and determine, so the glue is also prone to degradation with a temperature resulting in a drastic coupling decrease. Given all these factors and details, a 500±25μm Si substrate was Au-Au bonded to a $LiNbO_{3}$$\left(YXl\right)/128^{\circ }$ crystal wafer. Silicon was chosen as the substrate, as it is a well-studied material in the microelectronic industry, and thus, it remains an affordable and reliable alternative for scaling the micro-fabrication process, especially for MEMS scale devices, due to its compatibility with CMOS processes. The piezoelectric layer was thinned down by lapping, followed by micro-polishing steps until the thickness reached 27±2μm. The Au top electrodes were patterned by means of UV lithography, e-beam evaporation, and lift-off. The wafer bonding and fabrication steps were discussed in more detail in an earlier work [19]. The wafer was diced to form a cantilever and finally glued to a rigid substrate on the clamping side and made free to vibrate. The cantilever, 22 mm long and 5 mm wide, was wire-bonded and soldered onto the PCB to obtain a PEH device, as shown in Figure 1. Table 1 reports the geometrical dimensions of the cantilever, electrodes, and substrates. Figure 1a shows the cross-sectional layout of a PEH with a cantilever structure, consisting of a silicon substrate on which the piezoelectric $LiNbO_{3}$ is bonded. A proof mass with a weight of 2.3 g was added with the help of 3M double-sided tape to lower the operating frequency.

**Figure 2:**
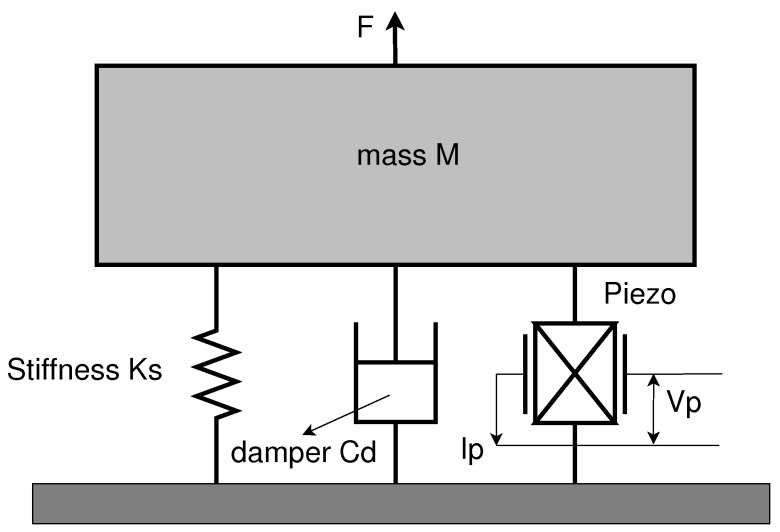
SDOF lumped parameter model for a piezoelectric transducer.

The cantilever structure of a PEH can be modeled as a Single Degree Of Freedom (SDOF) model. Figure 2 shows a system that includes a seismic mass, $m_{s}$, a spring with a stiffness constant, $K_{s}$, a viscous damper with a damping coefficient, $C_{d}$, and a vibrating base. A key challenge in designing a cantilever beam-type PEH consists of tuning the cantilever resonant frequency, $f_{r}$, to that of the forced vibration. Since most of the ambient mechanical vibrations available in the environment are less than 300 Hz, it is advantageous to set the operating frequency of the PEH in this regime. A simple and commonly used tuning method is the addition of a lumped-proof mass attached to the free end of the cantilever beam. In the PEH structure, the proof mass helps homogenize the strain distribution within the piezoelectric layer. Furthermore, adding a proof mass allows for increasing the squared global electro-mechanical coupling, $k^{2}$.

The seismic mass, $m_{s}$, is represented in Equation (1) as a function of the mass, $m_{p}$, of the proof mass and the mass, $m_{c}$, of the cantilever [20].

(1) $$m_{s}\;=\;m_{p}\;+\;0.23\cdot m_{c}$$

Equation (2) shows the relationship between the resonance frequency, $f_{r}$, of the cantilever and the proof mass, $m_{p}$ [21].

(2) $$f_{r}=\frac{1}{2\pi }\cdot \sqrt{\frac{K_{s}}{m_{p}\;+\;0.23\cdot m_{c}}}$$

In this work, we chose a resonance frequency, $f_{r}$, close to 200 Hz to include most of the ambient mechanical vibrations. Equation (3) shows the spring stiffness, $K_{s}$, as a function of the beam width, *w*, the thickness, *t*, the length, *l*, and Young’s modulus, *Y*.

(3) $$K_{s}=\frac{Y\cdot w\cdot t^{3}}{4\cdot l^{3}}$$

**Figure 3:**
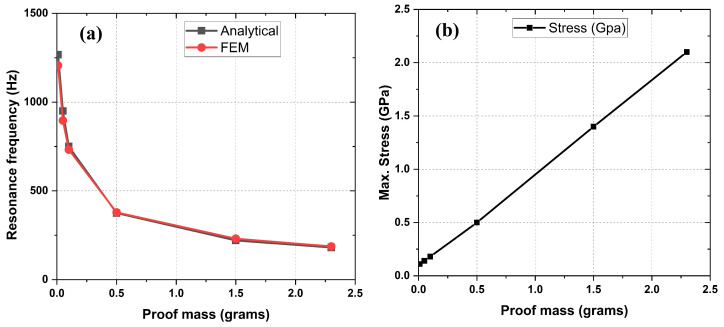
(**a**) Resonance frequency, $f_{r}$, vs. proof mass weight, $m_{p}$. (**b**) Maximum developed stress vs. proof mass weight, $m_{p}$.

Eigen-frequency analysis was performed using the Finite Element Method (FEM) with the Comsol Multiphysics software to obtain the resonance frequencies, $f_{r}$, and stress distribution in the PEH for various proof mass weights. A 2D, unimorph, cantilevered beam structure with the specified geometry in Table 1 was considered for eigen-frequency analysis. The materials and their properties were then assigned to the designed geometry. The substrate Si was assumed to be a linear elastic material that bends according to the stress produced. A body load was applied to the whole system, assuming a constant force per unit volume. One end of the beam is fixed so that the other end can vibrate about the mean position. Figure 3 shows the variation of the resonance frequency, $f_{r}$, of the PEH with the weight of the proof mass, $m_{p}$, as determined via analytical Equation (2) to be in good agreement with the FEM results. Figure 3a reveals that the resonance frequency, $f_{r}$, decreases sharply from 1.26 kHz to 187 Hz when the proof mass increases from 0.01 g to 2.3 g. Therefore, by selecting a proof mass of 2.3 g, the PEH resonance frequency, $f_{r}$, is significantly lowered to a value close to 200 Hz. To ensure the mechanical reliability of the PEH structure, it is necessary to ensure that the stress developed inside the cantilever beam falls within the range of the breaking stress of the substrate material (Si) and lithium niobate layer. Figure 3b shows the maximum stress distribution inside the cantilever beam, determined via FEM while varying the weight of the proof mass from 0.01 g to 2.3 g at a constant acceleration of 1 g. The graph reveals that the maximum stress of ≈2 GPa, developed at the bottom corner of the Si cantilever, is within the limit of the breaking stress or maximum stress limit of 3 GPa in Si [22,23]. Therefore, the proof mass was built with a symmetrical cube of tungsten weighing 2.3 g with a side dimension equal to the width of the PEH, providing a good trade-off between the lowering of the resonant frequency and a sufficient margin with respect to the maximum allowable stress in the material. Figure 3 shows that the device can operate below 2 g acceleration without a breakdown, i.e., it is adequate for energy harvesting from the ambient mechanical vibration present in the environment.

## 3. Estimation of Electro-Mechanical Properties

**Figure 4:**
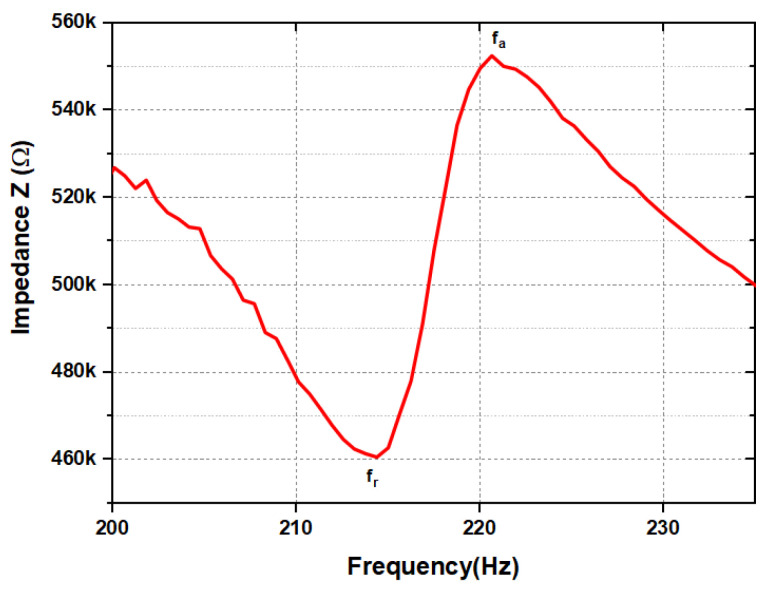
Anti-resonant frequency, $f_{a}\;=\;220\;$Hz at Zmax = 550 kΩ, and resonant frequency, $f_{r}\;=\;214\;$Hz at Zmin = 460 KΩ.

Even though, at the PEH resonance frequency, the input energy to the system is the maximum, significant losses still occur. To account for such losses of piezoelectric materials, the mechanical quality factor, $Q_{m}$, was used as a figure of merit. The measurement of the electrical impedance of the device enables the analysis of the electro-mechanical resonance frequencies and characteristics of the structure. Figure 4 shows the experimental results of the measurements of the resonance, $f_{r}$, and anti-resonance, $f_{a}$, frequencies of the PEH that correspond, respectively, to electrical short and open circuit conditions. The measurement of the electro-mechanical resonance frequencies $f_{r}$ and $f_{a}$ was performed using an impedance analyzer and by measuring the PEH electrical impedance while sweeping the frequency. The impedance of the device, over a range of frequencies, was measured with the impedance analyzer HIOKI-IM3570. A sinusoidal signal with a low AC amplitude of 1 V was supplied to the device to measure the voltage and current on the electrodes and indirectly evaluate the impedance.

Using Equation (4) to calculate the resonance quality factor, $Q_{m}$, where $f_{1}\;-\;f_{2}$ is the −3 dB bandwidth of the impedance curve around the resonance frequency, $f_{r}$, reveals that the measured quality factor of the PEH is $Q_{m}$ = 44.

(4) $$Q_{m}=\frac{f_{1}-f_{2}}{f_{r}}$$

### Electro-Mechanical Coupling Coefficient

The electro-mechanical coupling coefficient, *K*, is a dimensionless figure of merit that reveals the energy conversion ability of a material between the electrical and mechanical domains. In addition, higher electro-mechanical coupling implies that the stress is evenly distributed on the piezoelectric material, which allows the piezoelectric material to generate higher electric power with lower peak stress [24]. Among different definitions of the piezoelectric coupling coefficient, the most commonly used forms include the squared global electro-mechanical coupling coefficient, $k_{m}^{2}$, the squared piezoelectric material coupling factor, $k_{t}^{2}$, and the squared effective electro-mechanical coupling coefficient, $k_{eff}^{2}$ [25]. Their values are nearly the same for relatively low coupled devices. Hence, they are often used in an interchangeable way for devices with coupling below 5%. However, the difference between them becomes significant for large coupling cases. From the measurement of $f_{r}$ and $f_{a}$, it is possible to derive their values using Equations (5)–(7).

The global squared coupling coefficient $k_{m}^{2}$ was determined to be 0.06 from Equation (5):

(5) $$k_{m}^{2}=\frac{f_{a}^{2}-f_{r}^{2}}{f_{a}^{2}}$$

The squared electro-mechanical coupling factor, $k_{t}^{2}$, was calculated to be 0.08 using Equation (6).

(6) $$k_{t}^{2}=k_{31}^{2}=\frac{\pi }{2}\frac{f_{r}}{f_{a}}\tan\left[\frac{\pi }{2}\left(1-\frac{f_{r}}{f_{a}}\right)\right]$$

Moreover, the squared effective piezoelectric coupling coefficient, $k_{eff}^{2}$, was determined to be 0.07, and it can also be defined as in Equation (7):

(7) $$k_{eff}^{2}=\frac{k_{m}^{2}}{1-k_{m}^{2}}$$

The measured value of $k_{eff}^{2}=0.07$ represents a significant improvement compared to previous reported values, $k_{eff}^{2}=0.013$, for lithium niobate, $\left(YXlt\right)\;/\;36^{\circ }/\;90^{\circ }$, orientation devices [19], and the theoretically estimated piezoelectric material coupling coefficient value of lithium niobate, $\left(YXlt\right)\;/\;128^{\circ }/\;90^{\circ }$, orientation [18]. The energy harvesting Figure of Merit (FoM) of a PEH can be defined using Equation (8), and it was estimated to be FoM=3 in our present work.

(8) $$FoM=k_{eff}^{2}\cdot Q_{m}$$

These results are comparable with lead-based piezoelectric materials, thus encouraging to use of lead-free materials to design “green” and wide-band PEH devices.

## 4. Equivalent Circuit SPICE Model and Analysis of PEH

**Figure 5:**
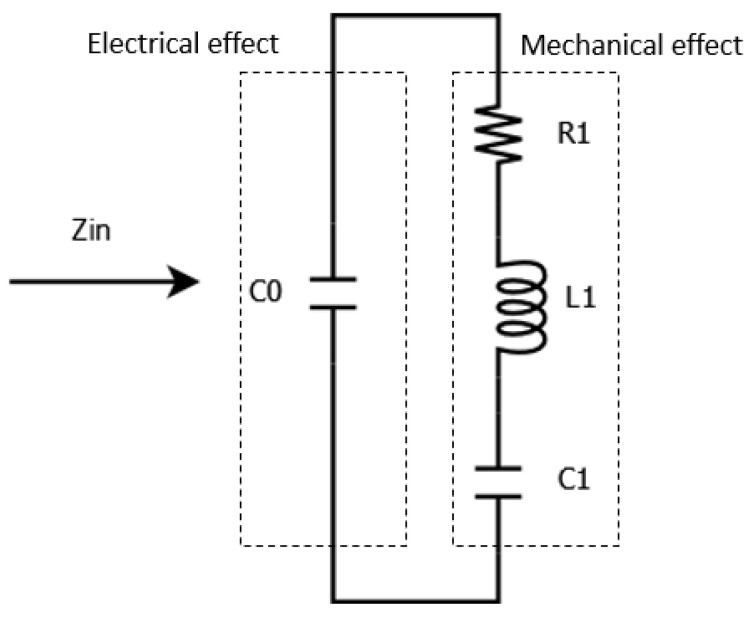
Van Dyke model of the electrical side determined from impedance analysis.

The equivalent circuit models with an overall impedance $Z_{in}$ can be used for the analysis and design of piezoelectric systems [26,27]. Understanding the characteristics of piezoelectric harvesters enables one to optimize the system’s performance. Thus, it is desirable to have a way to determine the electrical equivalent circuit parameters of a given transducer. The electrical circuit parameters of the PEH in the Butterworth–Van Dyke model [28] were estimated using the impedance measurements shown in Figure 5 [27].

The Butterworth–Van Dyke electrical model of a piezo is a simplified example of a coupled electromechanical system. The mechanical behavior of the mass-spring system (corresponding to the piezo with mass, stiffness, and damping) causes an electrical effect modeled with an inductance, $L_{1}$, a capacitance, $C_{1}$, and a resistive damping term, $R_{1}$. These are coupled with the electrical capacitance, $C_{0}$, of the piezo.

The Mason’s model of the harvester was also derived. Mason’s model decouples the mechanical and electrical sides of the harvester, and thus, it is made possible to predict the electrical output with various load resistance conditions and also to optimize the power harvested and the conditioning circuit. The electro-mechanical coupling is expressed in the coupling factor α, which has the dimension of N/V. The parameters describing the electrical side are the capacitance, $C_{0}$, and the parallel resistor, *R*, which represent dielectric losses in the piezoelectric material. The elements $R_{m}$, $L_{m}$, and $C_{m}$ correspond to a mechanical mass–spring system, for which $R_{m}$ represents the mechanical dissipation, $L_{m}$ is the mass, and $C_{m}$ is the compliance of the material. The two parts (mechanical and electrical sides) are connected via a transformer converting the electrical energy to mechanical energy, and vice versa. Here, the mechanical branch of Mason’s form was derived from the Butterworth–Van Dyke topology. For the mechanical side, the conversion factor α was used in order to obtain the mechanical parameters ($R_{m}$, $L_{m}$, and $C_{m}$) in the electrical equivalent form ($L_{m}=\alpha^{2}\cdot L_{1}$, $R_{m}=\alpha^{2}\cdot R$, $1/C_{m}=C_{1}/\alpha^{2})$ [26]. To determine the parameter α, the PEH was supplied with a sinusoidal voltage with a peak amplitude, $V_{p}$, in an open circuit condition. The vibration amplitude peak, *x*, was measured using a laser displacement sensor setup, and α was measured using Equation (9).

(9) $$\alpha =\frac{C_{0}\cdot V_{p}}{x}$$

The parallel resistance of 9 MΩ, was included in the full Mason’s model to indicate the internal leakage losses. The extracted parameters are given in Table 2.

**Figure 6:**
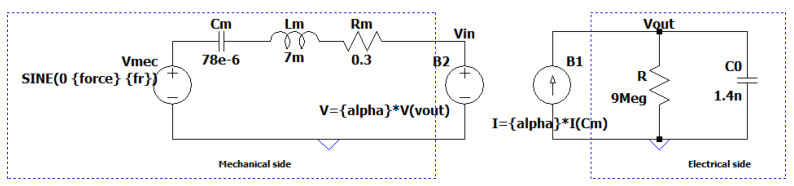
LT-Spice model for the Mason’s electro-mechanical equivalent circuit. For stability and to avoid the effect of primary and secondary coils, the transformer was replaced with an interdependent voltage-controlled voltage source on the mechanical side and a current-controlled current source on the electrical side.

The electro-mechanical model of the PEH was implemented as an equivalent electric circuit with the LT-Spice simulation environment. The SPICE model permits the analysis of a transducer, particularly around its resonance frequencies, and it is useful in the design of electrical impedance matching circuits to obtain the maximum power transfer. The validity of the extracted equivalent circuit of the device, represented in Figure 6, was ascertained by performing impedance analysis in LT-Spice using the model parameters listed in Table 2 and comparing it with the experimental impedance analysis results obtained using an impedance analyzer for the given frequency range from 100 Hz to 300 Hz.

**Figure 7:**
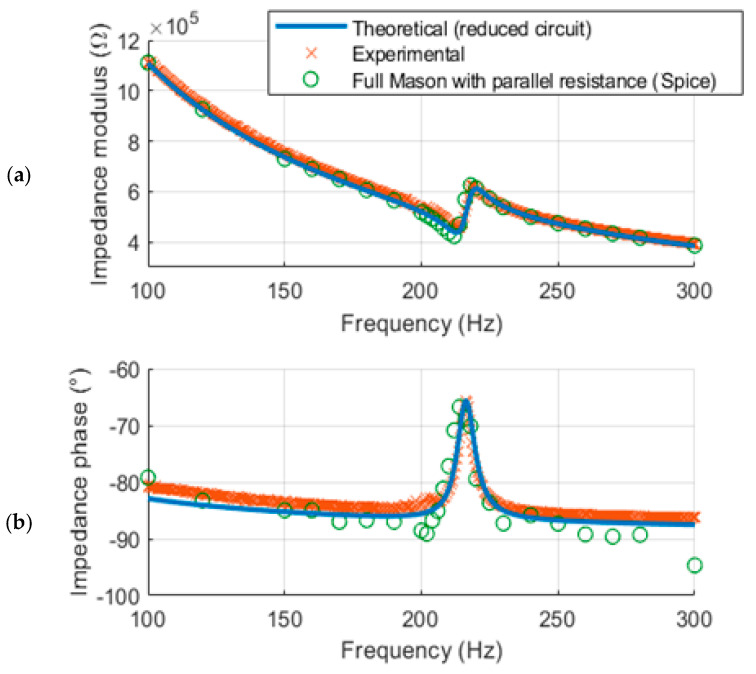
(**a**) Impedance modulus and (**b**) phase from theoretical (reduced circuit), experimental, and full Mason’s model in Spice.

The LT-Spice simulation of the full Mason’s model closely agreed with the theoretical impedance of the reduced circuit, as well as the experimental results obtained from the impedance analyzer, as shown in Figure 7.

## 5. Optimal Load Resistance for Maximum Output Power

According to the power transfer theorem, the maximum amount of power output from a source occurs when the external electrical impedance matches the internal impedance of the source. The Root Mean Square (RMS) power output of the device was calculated via Equation (10) by measuring the RMS voltage ($V_{RMS}$) across an external load resistance $R_{load}$.

(10) $$P_{RMS}=\frac{V_{RMS}^{2}}{R_{load}}$$

**Figure 8:**
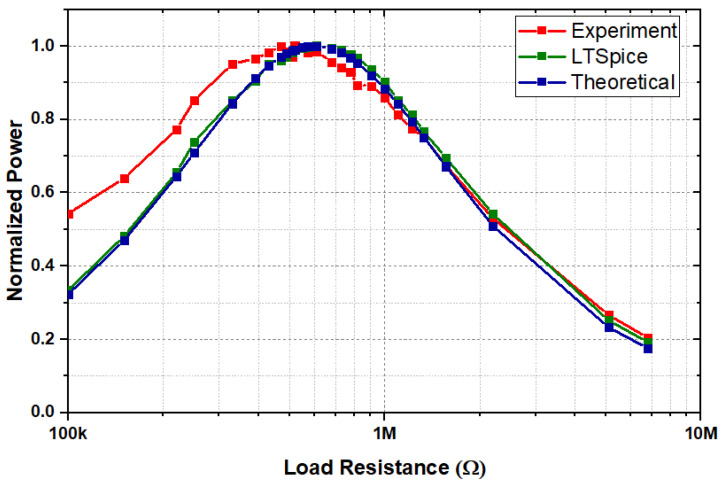
Normalized harvested power vs. load resistances for different models.

The PEH was excited at resonance, and the optimal load corresponding to the maximum power was determined experimentally by varying the load resistance (10). Furthermore, the optimal load at resonant frequency $f_{r}$ was simulated via the analysis of the full Mason’s equivalent circuit (Figure 5) using LT Spice at different load resistances, $R_{load}$. The optimal load resistance, $R_{opt}$, at which the maximum power, $P_{max}$, was delivered was determined theoretically and compared with the experimental results and SPICE simulations, as depicted in Figure 8.

In AC conditions, the theoretical optimal load resistance, $R_{opt}$, of the piezoelectric element can be expressed as in Equation (11)

(11) $$R_{opt}=\frac{1}{C\cdot \omega }$$

where ω is the angular frequency, and C is the capacitance of the piezoelectric element. The load resistance, $R_{load}$, vs. the normalized power for the simulation, experimental, and theoretical results is shown in Figure 8. The LT Spice simulations and experimental results closely agreed with the theoretical model of the harvester with an optimal load ($R_{opt}$) close to 520 kΩ. There was a slight deviation in the measured experimental optimal load with the theoretical value, apparently due to changes in damping factors during measurement.

## 6. Test Results and Analysis

**Figure 9:**
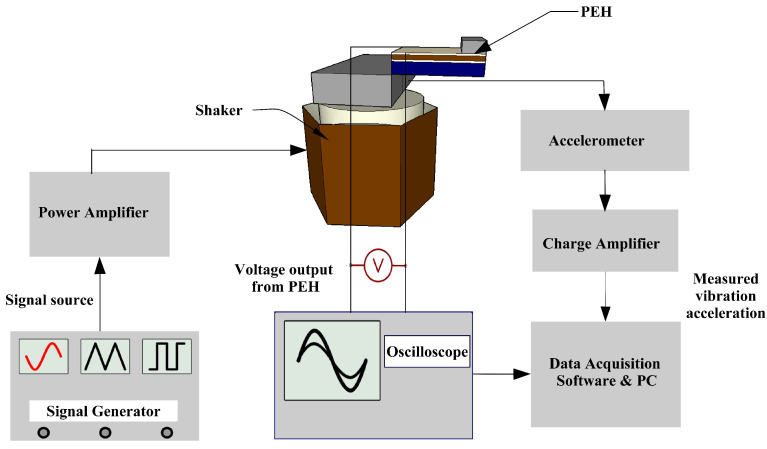
Schematic of the experimental setup with the shaker.

The open circuit voltage, $V_{oc}$, response and the output power, $P_{out}$, were measured by mounting the device on a shaker setup. The system consists of a shaker (electrodynamic shaker—Data Physics), an accelerometer (PCB Piezotronics 355B04), a 100 W power amplifier, an oscilloscope, a signal generator, and a charge amplifier, along with data acquisition software to display the output on a computer. The schematics of the experimental setup are shown in Figure 9. An accelerometer was mounted on the shaker close to the PEH device for the acceleration measurement. The sinusoidal signal from the signal generator was amplified via the power amplifier that set the vibration amplitude and frequency of the shaker. The output voltage generated via the PEH, which depends on the signal generated from the signal generator, was observed through the oscilloscope and identified via the software. Simultaneously, the output signal from the acceleration sensor was amplified via the charge amplifier and observed on a monitoring computer.

**Figure 10:**
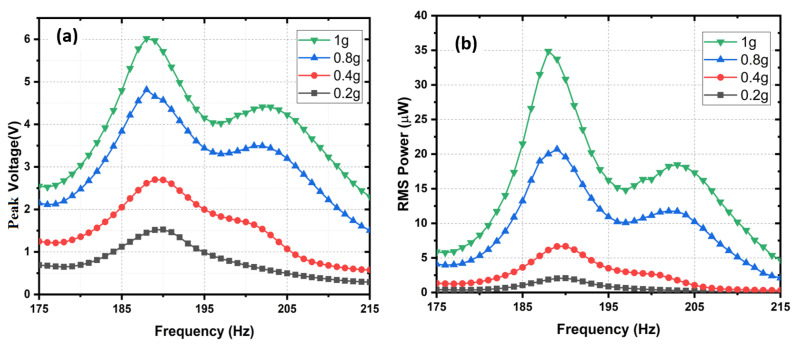
(**a**) Peak voltage *V*; (**b**) RMS power $P_{RMS}$.

The output peak voltage, $V_{p}$, and the RMS power, $P_{rms}$, in the frequency range from 175 Hz to 215 Hz and at different acceleration values from 0.2 g to 1 g were measured at an optimal load of 520 kΩ and illustrated in Figure 10, respectively, in graphs (a) and (b). The graphs show that $V_{p}$ and $P_{rms}$, at an acceleration level of 1 g, and the optimal load were close to 6 V and 35 μW, respectively.

**Figure 11:**
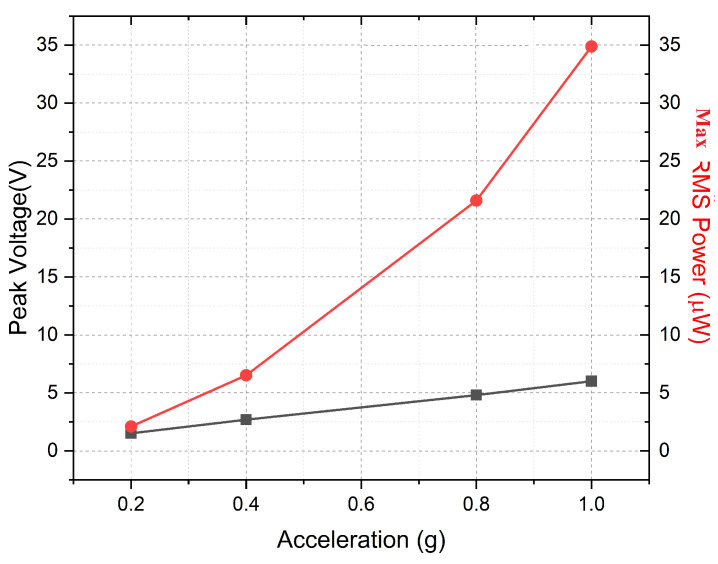
Experimental results: peak voltage α·g and maximum RMS power $\left(\alpha \cdot g^{2}\right)$ in the acceleration range from 0.2 g to 1 g at an optimal load.

Figure 11 shows the output peak voltage, $V_{p}$, and the RMS power, $P_{rms}$, at a resonance frequency and the optimal load in the acceleration range from 0.2 g to 1 g.

Since the system was designed to have sufficiently large $k_{eff}^{2}\cdot Q_{m}$, it can be observed from Figure 10 that, for optimal resistive loads ($R_{load}$), two maxima peaks for power exist, separated by a dip in the curve. Therefore, this effect of high coupling is a clear advantage, as it can be used to extract energy from a broad spectrum of input frequency. The overall RMS and peak power density (normalized with the input acceleration and area) of the PEH at 0.2 g were estimated to be 60 μW/cm^2^/g^2^ and 120 μW/cm^2^/g^2^, respectively.

In several application scenarios, the input vibration spectrum of the PEH fluctuates. For instance, the vibration frequency range of a water pump in a combined heat and electric power plant varies in the range of 210 Hz to 219 Hz (≈5%) as the speed of the pump changes [29].

Therefore, the power bandwidth, BW, is an important parameter to determine the operating frequency range. It is defined by the half-power cut-off frequencies $f_{B1}$ and $f_{B2}$ [14] (for which $P_{PEH}\;=\;0.5\cdot P_{PEH\_max}$), as in Equation (12):

(12) $$BW=\;\frac{f_{B1}-f_{B2}}{f_{0}}$$

with $f_{0}$ corresponding to the frequency at the maximum power, $P_{PEH_{m}ax}$. In this work, the reported device exhibited a power BW of ≈10.8% at resonance. The performances of the fabricated prototypes and the comparison to the state-of-the-art devices in terms of normalized power density and bandwidth are listed in Table 3, which shows that the proposed device competes with other systems, hence providing an attractive lead-free alternative in the framework of the environmentally friendly power systems.

## 7. Power Harvesting Circuit for an IoT Node

**Figure 12:**
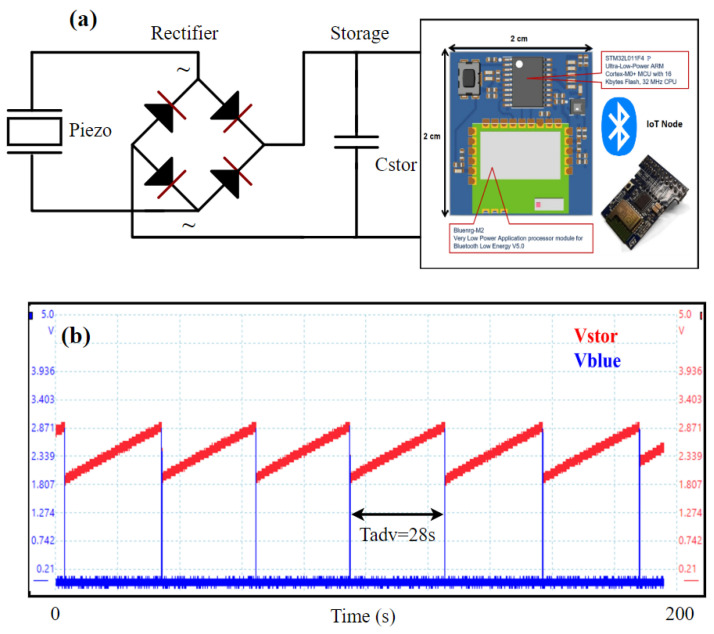
(**a**) PEH powering an IoT node with a full bridge rectifier and a storage capacitor. (**b**) Voltage profile ($V_{stor}$)across the capacitor $C_{stor}$ after rectification at 1 g acceleration while driving an IoT device; $V_{blue}$ is the voltage across the BLE radio during a data transmission event, and Tadv is the advertising time of the BLE beacons.

IoT sensor nodes require a stabilized DC voltage, and the output of the PEH is inherently an AC voltage that needs to be rectified, filtered, and regulated to provide usable output power. Several types of electronic interfaces have been proposed in the literature, such as SSHI, Hybrid SSHI, and DSSH standard models [33,34,35] for collecting the DC output based on the $k_{eff}^{2}\cdot Q_{m}$ of the device [36]. In this work, the $k_{eff}^{2}\cdot Q_{m}$ was determined to be over 3, and we chose the standard rectification scheme employing a full bridge. The power generated via the PEH is not sufficient to be used for continuous applications. However, since several applications involving WSNs operate in a discontinuous mode, it is enough to use a power harvesting circuit to generate energy in a burst mode for a short time [37]. This battery-free concept was implemented by initially storing the harvested energy in a storage capacitor ($C_{stor}\;=220\;\mu$F) and then supplying the stored energy in an IoT device from the capacitor, as shown in Figure 12a. In this way, we did not need bulky batteries, as the energy in the capacitor was replenished via the harvester.

The voltage profile ($V_{stor}$) across the capacitor $C_{stor}$ during the charging process is shown in Figure 12b. When driven at the acceleration of 1 g, the harvester can fully charge the $C_{stor}$ capacitor to its rated voltage of 6 V in nearly 500 s. The PEH can power a wireless IoT node based on a Bluetooth Low Energy (BLE) radio at acceleration as low as 0.5 g, using the energy stored in the capacitor. Further, the IoT node can send data via BLE in advertising mode with a duty cycle of 28 s at an acceleration of 1 g, as shown in Figure 12b.

## 8. Conclusions

The fabrication and characterization of a lead-free, piezoelectric, broadband micro-harvester device working at low-frequency vibrations based on lithium niobate were undertaken. The PEH equivalent circuit with global parameters was determined using Mason’s model. The impedance of the electro-mechanical equivalent circuit was simulated with Spice and found to be in good agreement with the experimental results obtained using an impedance analyzer. The optimal load, $R_{load}$, for the maximum output power was determined via experimental, LT-Spice, and theoretical analysis and determined to be close to 520 kΩ. The experimental simulation and theoretical values were in close agreement, and they indicate the validity of the three approaches. At this optimal load and a typical 1 g input acceleration, the harvester prototype can deliver/generate a maximum output voltage of 6 V and RMS power of 35 µW, sufficient for low-power IoT sensor nodes. In addition, the output voltage from the PEH exceeded the forward conduction threshold voltage for a diode over a wide broadband frequency range, which is promising and addresses issues in many real-world applications where the ambient vibration frequency changes. The harvester provides an excellent power bandwidth of over 10 percent, thanks to the relatively high device electro-mechanical coupling, comparable with lead-based materials. The overall RMS PEH power density was estimated to be 70 µW/cm^2^/g^2^ at 0.2 g. Thanks to the relatively higher power density, the harvester is able to power a wireless IoT sensor node at accelerations as low as 0.5 g, showing the application potential of such green PEH devices. Also, this work highlights that, by adopting an “install and forget approach” through harvesting ambient vibration energy using lead-free piezoelectric energy harvesters, we can avoid using primary batteries and the related impact on the environment. Finally, this work on lithium niobate as a valid candidate for ambient vibration energy harvesting for IoT applications projects a positive outlook in electronics for lead-free, green alternatives using piezoelectric materials. Ways to improve the performance of the devices, such as optimizing the thickness of the piezoelectric layer or the fabrication of bimorph structures, will be explored in future studies.

## Figures and Tables

**Table 1:** Cantilever, top electrode, and substrates geometrical dimensions of the Au/$LiNbO_{3}$/Au/Si PEH.

Cantilever Length l (mm)	Cantilever Width *w* (mm)	Electrode Length (mm)	Electrode Width (mm)	Silicon Thickness *t_s_* (μm)	${\mathrm{LiNbO}}_{3}$ Thickness *t_p_* (μm)	Proof Mass Width (Grams)
22	5	22	4.9	500	27	2.3

**Table 2:** Extracted lumped parameters.

$C_{0}$	$C_{1}$	$C_{m}$	$R_{1}$	R	$R_{m}$	$L_{1}$	$L_{m}$	α (N/V)
1.4 nF	18.8 pF	78 uF	1.4 MΩ	9 MΩ	300 mΩ	29 kH	7 mH	4.9 × 10^−4^

**Table 3:** Comparison of the broadband performance of $LiNbO_{3}$ (this work) with state-of-the-art, highly coupled lead-based materials.

Piezoelectric Material	RMS Power (µW)	Acceleration (g)	$f_{r}$ (Hz)	$Q_{m}$	$k_{\mathrm{eff}}^{2}$	Bandwidth
PZT [30]	670	0.56	253	50	15.4%	7.5%
PZT [24]	90	0.4	152	95	6.7%	10.5%
PZT [14]	0.39	0.02	32	85	11.3%	7.8%
PMN-PT [14]	0.58	0.019	29	130	16%	10.1%
PMN-PT [31]	32.7	0.02	31	58	17.6%	8.4%
PZN-PT [14]	1.3	0.017	32	91	16.4%	11.3%
PVDF [32]	112.8	0.5	34.4	17.2	3.6%	9.5%
$LiNbO_{3}$ (This work)	35	1	187	44	7.2%	10.8%

## Data Availability

Data are contained within the article.
